# Mir-34a-5p Mediates Cross-Talk between M2 Muscarinic Receptors and Notch-1/EGFR Pathways in U87MG Glioblastoma Cells: Implication in Cell Proliferation

**DOI:** 10.3390/ijms19061631

**Published:** 2018-05-31

**Authors:** Maria Di Bari, Valeria Bevilacqua, Antonella De Jaco, Pietro Laneve, Roberta Piovesana, Laura Trobiani, Claudio Talora, Elisa Caffarelli, Ada Maria Tata

**Affiliations:** 1 Department of Biology and Biotechnology “C. Darwin”, Sapienza University of Rome, P.le Aldo Moro, 5, 00185 Rome, Italy; maria.dibari@uniroma1.it (M.D.B.); bevilacqua@ingm.org (V.B.); antonella.dejaco@uniroma1.it (A.D.J.); roberta.piovesana@uniroma1.it (R.P.); laura.trobiani@uniroma1.it (L.T.); 2 Institute of Molecular Biology and Pathology, National Research Council of Italy, c/o Sapienza University of Rome, P.le Aldo Moro, 5, 00185 Rome, Italy; pietro.laneve@uniroma1.it; 3 Department of Molecular Medicine, Sapienza University of Rome, Viale Regina Elena, 291, 00161 Rome, Italy; claudio.talora@uniroma1.it; 4 Research Center of Neurobiology “Daniel Bovet”, Sapienza University of Rome, P.le Aldo Moro, 5, 00185 Rome, Italy

**Keywords:** M2 muscarinic receptors, glioblastoma, Notch-1, EGFR, mir-34a-5p, p53

## Abstract

Glioblastoma (GBM) is the most aggressive human brain tumor. The high growth potential and decreased susceptibility to apoptosis of the glioma cells is mainly dependent on genetic amplifications or mutations of oncogenic or pro-apoptotic genes, respectively. We have previously shown that the activation of the M2 acetylcholine muscarinic receptors inhibited cell proliferation and induced apoptosis in two GBM cell lines and cancer stem cells. The aim of this study was to delve into the molecular mechanisms underlying the M2-mediated cell proliferation arrest. Exploiting U87MG and U251MG cell lines as model systems, we evaluated the ability of M2 receptors to interfere with Notch-1 and EGFR pathways, whose activation promotes GBM proliferation. We demonstrated that the activation of M2 receptors, by agonist treatment, counteracted Notch and EGFR signaling, through different regulatory cascades depending, at least in part, on p53 status. Only in U87MG cells, which mimic p53-wild type GBMs, did M2 activation trigger a molecular circuitry involving p53, Notch-1, and the tumor suppressor mir-34a-5p. This regulatory module negatively controls Notch-1, which affects cell proliferation mainly through the Notch-1/EGFR axis. Our data highlighted, for the first time, a molecular circuitry that is deregulated in the p53 wild type GBM, based on the cross-talk between M2 receptor and the Notch-1/EGFR pathways, mediated by mir-34a-5p.

## 1. Introduction

Glioblastoma multiforme (GBM) is the most common primary brain tumor and is considered the most aggressive and malignant human cancer [1]. Accordingly, GBM is extremely invasive and shows high ability to infiltrate through the brain parenchima. Moreover, this tumor shows high chemo- and radio-resistance, making the identification of new molecular targets for cell growth and survival relevant for GBM therapy. The most frequent signaling pathways dysregulated in GBMs are Notch and epidermal growth factor receptor (EGFR) [2]. The Notch pathway appears largely involved in the outcome or progression of several tumors [3,4]. The Notch proteins (Notch 1–4) are transmembrane receptors produced as long polypeptides that are activated by several proteolytic cleavages. In particular, the cleavage operated by the gamma-secretase complex releases the Notch intracellular domain (NICD), which activates the family of basic helix–loop–helix (bHLH) transcriptional repressors. Among them, the Hes/Enhancer of split (Hes 1–7) and Hey (Hey 1–2) are able to influence cell proliferation and differentiation during nervous system development [5]. Aberrant expression of proteins involved in the Notch cascade may play relevant roles in glioma development. It has been demonstrated that the knockdown of Notch-1 or the inhibition of its activity in glioma cell lines led to cell cycle arrest, accompanied by decreased cell proliferation and increased cell death [6,7].

The EGFR pathway has been found to be frequently over-expressed or hyper-activated in a number of epithelial tumors as well as in GBM [8,9]. Alterations in EGFR signaling can lead to apoptosis or enhanced proliferation, angiogenesis and necrosis, suggesting a strong correlation between dysregulated receptor activity and the pathobiology of many cancers. Interestingly, a large body of evidence indicates that the Notch pathway is intimately coupled to EGFR or its downstream targets, both in development and in cancer [10].

Accordingly, the activation of AKT and/or RAS signaling downstream of EGFR induces Notch-1 expression, possibly by recruiting existing Notch-1 mRNA to polysomes and increasing its translation [11]. On the other hand, a direct link between Notch-1 and EGFR in gliomas has been also demonstrated by the ability of Notch-1 to control EGFR expression in a p53-mediated manner. In fact, it has been reported that Notch-1 inhibition causes a decrease of EGFR mRNA and protein levels [10].

Acetylcholine muscarinic receptors including five subtypes (M1–M5), are members of the G Protein–Coupled Receptors (GPCRs) [12]. These receptors are widely distributed both in the central and peripheral nervous system, and in several mammalian organs [13]. While in vitro and in vivo studies have indicated that the activation of M3 receptors enhanced tumor cell proliferation [14,15,16], we have demonstrated that the activation of M2 receptors, by arecaidine propargyl ester (APE) was able to arrest cell proliferation in GBM cell lines (U87MG and U251MG) and GB cancer stem cells [17,18,19]. Moreover, M2 receptor activation reduced cell survival, inducing oxidative stress and severe apoptosis [20]. Previous work from our group showed that blocking the M2 receptor functioning by pharmacological competition and silencing experiments resulted in the complete abolishment of APE effects, suggesting the specificity of the agonist action of APE on M2 receptors [18,19].

In the present study, we identified the mechanism underpinning the M2-mediated cytostatic effect by demonstrating that the activation of the receptor, in p53-wildtype GBM cells, triggered mir-34a-5p expression, down-regulating Notch-, ,and affecting cell proliferation.

## 2. Results

### 2.1. M2 Receptor Activation Modulates Notch-1 Expression

**Figure 1:**
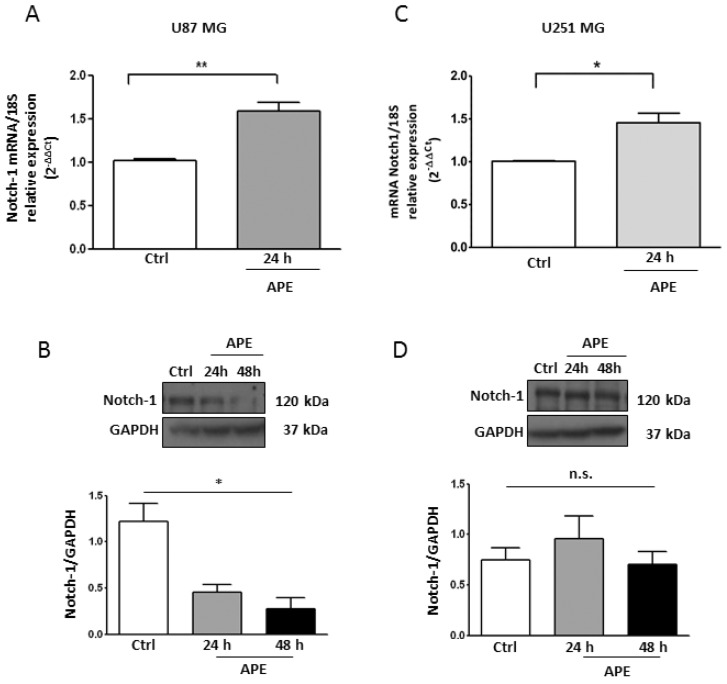
Notch-1 Expression in GBM cell lines. Real time RT-PCR and Western blot analysis (**A** and **B**, respectively) for Notch-1 in U87MG and in U251MG cells (**C** and **D**, respectively) cultured in the absence or presence of 100 μM APE for 24 and 48 h. Representative blots are shown from three independent experiments. GAPDH was used as the internal reference protein (* *p* < 0.05, ** *p* < 0.01).

*Notch-1* appears to act as an oncogene in GBM cells. Accordingly, the Notch pathway is over-expressed in the majority of the GBM lines and primary cells, contributing to cell transformation, growth, and survival [6]. To investigate the mechanism underlying the decrease in cell proliferation mediated by the M2 receptor, we chose two GBM cell lines, U87MG and U251MG, which mimic wild type or mutant p53 GBMs, respectively [18]. Quantitative real time PCR (qRT-PCR) analyses in U87MG cells indicated that Notch-1 mRNA significantly increased after 24 h upon APE treatment (Figure 1A). Notably, the Notch-1 protein significantly decreased by about 60% (Figure 1B). In the U251MG cell line while the Notch-1 mRNA increased by about 50% after M2 receptor activation (Figure 1C), Notch-1 protein levels remained unchanged (Figure 1D).

### 2.2. M2 Receptor Activation Induces Mir-34a-5p Expression in U87MG Cells

**Figure 2:**
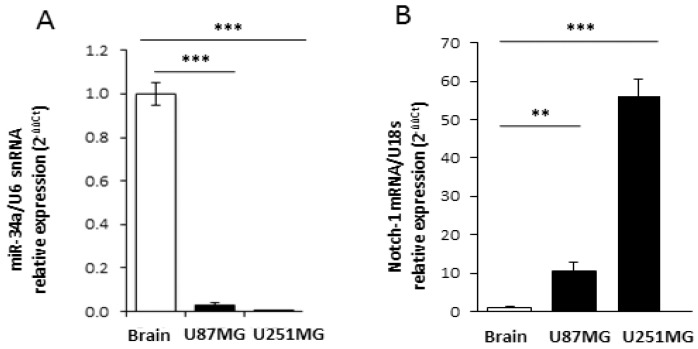
Expression of Notch-1 and miR-34a-5p in GBM cell lines and human brain. Real time RT-PCR analysis of miR-34a-5p (**A**) and Notch-1 (**B**) relative expression in U87MG or U251MG cell lines (black bars) compared to human normal brain (white bar). snRNA U6 and 18S were respectively used as the internal standard (** *p* < 0.01; ***p < 0.001; *t*-test).

**Figure 3:**
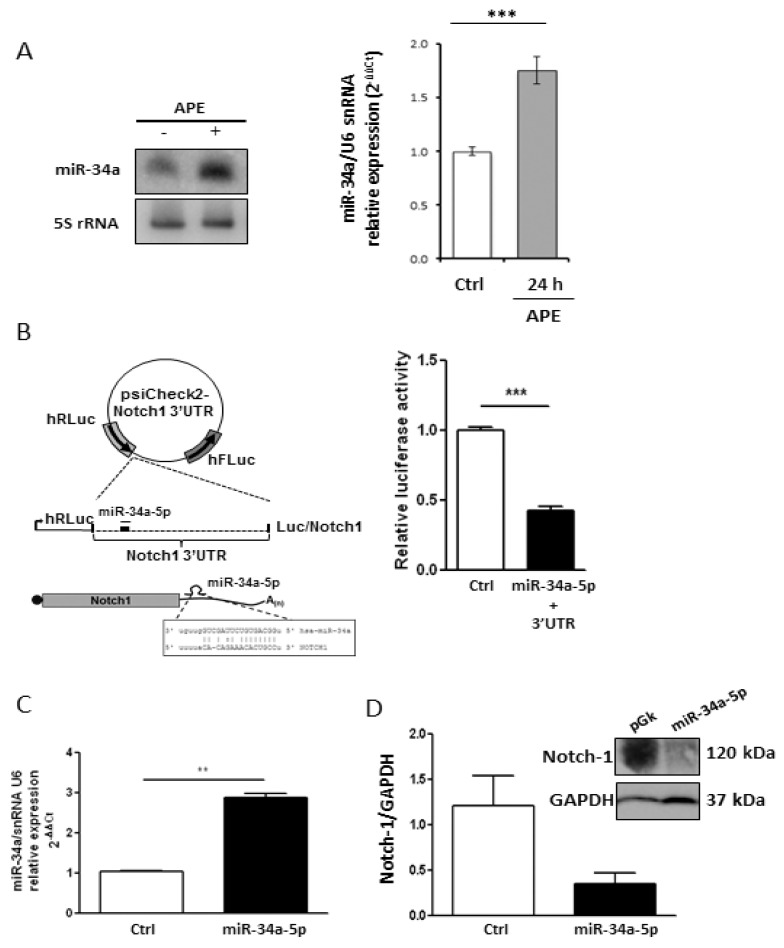
Analysis of Notch-1/miR-34-5p interaction. (**A**) Analysis of miR-34a-5p expression in U87MG cells, treated with 100 μM APE, by Northern blot (left) and real time RT-PCR (right) (*** *p* < 0.001 *t*-test); (**B**) Upper scheme: representation of Luc/Notch reporter construct. MiR is indicated as a thin line, miRNA response element as a thick line. Lower scheme: representation of 3′UTR region (with related sequences) binding mir-34a-5p. Right panel: luciferase activity (Renilla/Firefly ratio) of Notch-1 3′UTR reporter gene in HEK293 cells transfected for 48 h with the mir-34a-5p expressing vector or with empty vector used as control (Ctrl). Data are presented as mean ± SD from at least three different experiments. (*** *p* < 0.001 *t*-test); (**C**) mir-34a-5p over-expression after transfection in U87 cells (** *p* < 0.01 *t*-test); (**D**) Western blot analysis for Notch-1 levels in U87MG cells transfected with mir-34a-5p.

**Figure A1:**
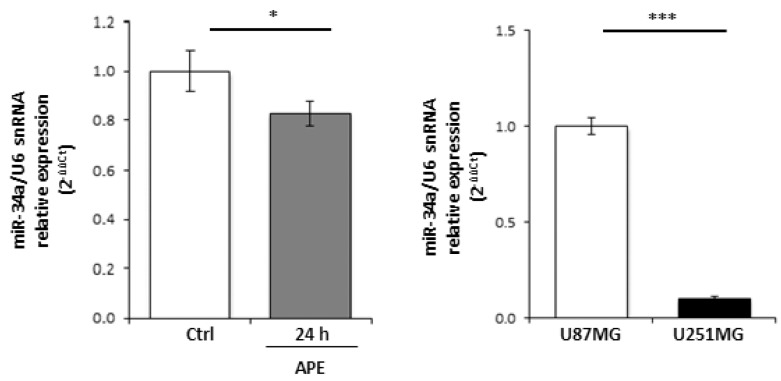
(**A**) Expression of miR-34-5p in GBM cell lines upon APE treatment. qRT-PCR (real time PCR analysis) expression analysis of miR-34-5p in U251MG untreated or treated for 24 h with 100 μM APE. (**B**) Relative expression of miR-34a-5p in U251MG cells versus U87MG cells. snRNA U6 was used as the internal standard (* *p* < 0.05; *** *p* < 0.001; *t*-test).

The relevant decrease of Notch-1 protein in APE-treated U87MG cells suggests the occurrence of a post-transcriptional regulation. Since microRNAs (miRNAs) negatively control gene expression at the post-transcriptional level, we investigated their possible implication in Notch-1 expression regulation upon APE treatment. Bioinformatics analysis using the miRNA prediction web tool microRNA.org [21] provided a list of putative miRNAs targeting Notch-1 3′UTR. Among these, mir-34a-5p was reported to be expressed at higher levels in wild type p53 than in the mutant GBM [22]. Furthermore, Notch-1 has already been validated as a *miR-34a-5p* target gene in several tumor histotypes [23] such as choriocarcinoma [24], breast cancer [25], and hepatocellular carcinoma [26]. We initially evaluated the levels of *miR-34a-5p* in both cell lines and in the normal brain. According to its role as an onco-suppressor in glioblastoma [23,27], we found that it was heavily downregulated in both cell lines when compared to the normal human brain (Figure 2A). Interestingly, messenger levels for Notch-1 were higher in GBM cell lines in comparison to the human normal brain (Figure 2B). Following treatment of both cell lines with APE, it showed that mir-34a-5p was significantly upregulated upon M2 receptor activation in U87MG cells as highlighted by the Northern blot (Figure 3A, left) and qRT-PCR (Figure 3A, right) analyses. Differently, it was expressed at lower levels in U251MG cells where it was not induced upon APE treatment (Appendix A Figure A1).

To verify the ability of mir-34a-5p to specifically interact with Notch-1 3′UTR in our cell system, a classic Luciferase (Luc) reporter assay was performed. HEK293T cells were co-transfected with a vector over-expressing mir-34a-5p and a reporter plasmid containing the portion of Notch-1 mRNA 3′-UTR, which includes the most conserved mir-34a-5p putative binding site according to Targetscan (release 7.1) fused to the Luc open reading frame (ORF) (Figure 3B, left). As shown in the histogram of Figure 3B (right), mir-34a-5p ectopic expression reduced the Luc activity by 50%, confirming that it may be involved in Notch-1 control. We validated this interaction in our cell system by over-expressing mir-34a-5p in the U87 cell line (Figure 3C) which showed that the endogenous Notch-1 protein levels, analyzed by Western blot, were strongly decreased after mir-34a-5p over-expression (Figure 3D).

### 2.3. M2 Receptor Activation Modulates Notch-2 and Hes-1 Expression

**Figure 4:**
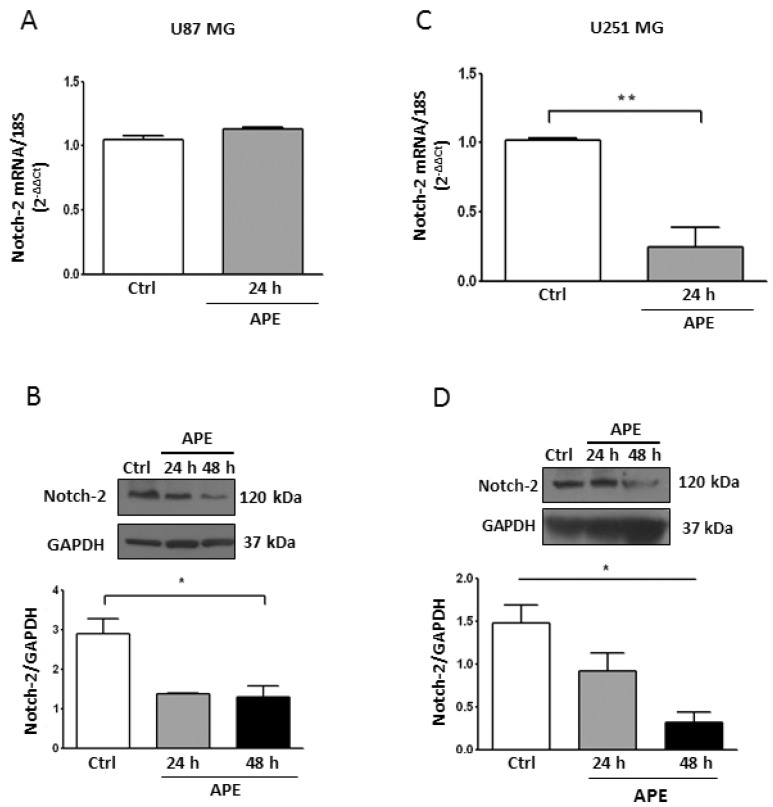
Notch-2 Expression in GBM cell lines. Real time RT-PCR and Western blot analysis (**A**,**B**, respectively) of Notch-2 in U87MG. Parallel analyses were performed in U251MG cells (**C**,**D**, respectively). Both lines were untreated or treated with 100 μM APE for 24 and 48 h. Representative blots are shown from three independent experiments. GAPDH was used as the internal reference protein. (* *p* < 0.05 One-way ANOVA test, ** *p* < 0.01 *t*-test).

We previously demonstrated that APE was able to inhibit cell proliferation in the U251MG cell line [17,18]. However, Notch-1 protein levels were not altered by the M2 agonist in these cells (Figure 1D), which prompted us to investigate whether the Notch-2 expression was also affected by APE treatment. We found that *Notch-2*, described as an additional oncogene in GBM [28], was negatively modulated by APE. While in the U87MG cell line, only the levels of the Notch-2 protein, but not the messenger, were reduced (Figure 4A,B); in the U251MG cells, both mRNA and protein levels decreased upon M2 agonist treatment (Figure 4C,D).

**Figure 5:**
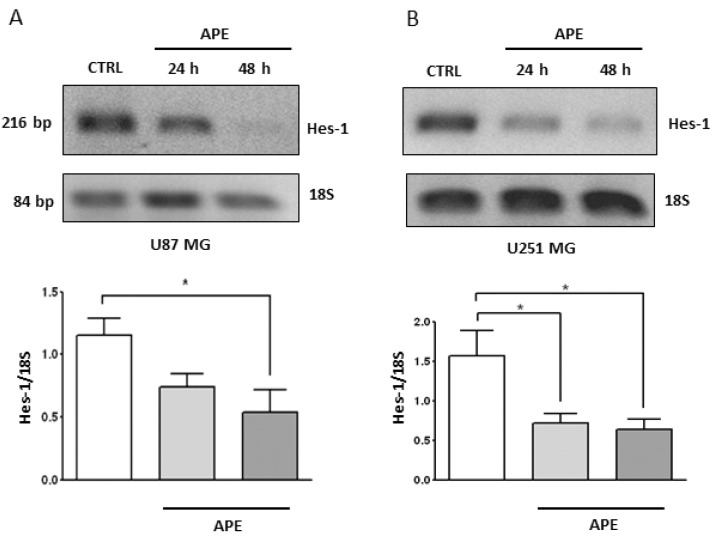
Hes-1 Expression in GBM cell lines. RT-PCR analysis of Hes-1 in U87MG (**A**) and U251MG (**B**) cells treated with 100 μM APE for 24 and 48 h. The graphs show the densitometric analysis of the bands normalized for the housekeeping 18S. The OD is the mean ± SEM of three independent experiments. (* *p* < 0.05; One-way ANOVA test).

To further investigate whether APE-mediated Notch downregulation may impact on the Notch pathway, the expression of Hes-1, which is one of the main transcription factors directly regulated by Notch [29] was analyzed. Hes-1 mRNA levels significantly decreased in both the U87MG (Figure 5A) and in U251MG cells (Figure 5B).

### 2.4. M2 Agonist Treatment Negatively Modulates EGFR Expression

Another pathway involved in GBM growth and survival is the EGFR signaling. To investigate whether M2 receptor activation also impacts on this pathway, we evaluated the EGFR mRNA and protein levels by qRT-PCR and Western blot analyses, respectively.

**Figure 6:**
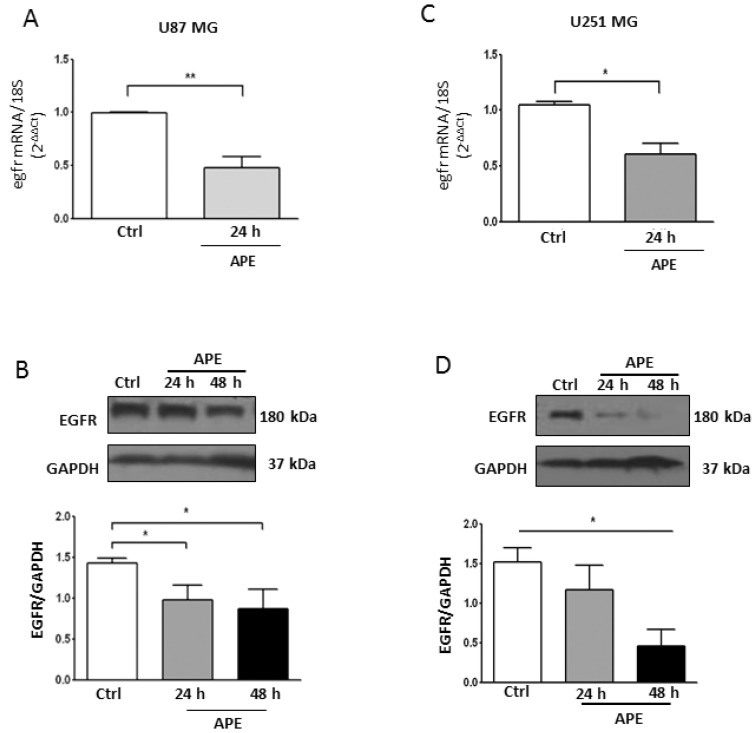
EGFR Expression in GBM cell lines. Real time RT-PCR and Western blot analysis (**A**,**B**, respectively) of EGFR in U87MG. Parallel analyses were performed in U251MG cells (**C**,**D**, respectively). Both lines were untreated or treated with 100 μM APE for 24 and 48 h. Representative blots are shown from three independent experiments. GAPDH was used as the internal reference protein. (* *p* < 0.05; ** *p* < 0.01; *t*-test and one-way ANOVA test).

As shown in Figure 6, M2 receptor activation caused a decrease of EGFR transcript and protein levels in both U87MG (Figure 6A,B) and U251MG (Figure 6C,D) cell lines.

**Figure 7:**
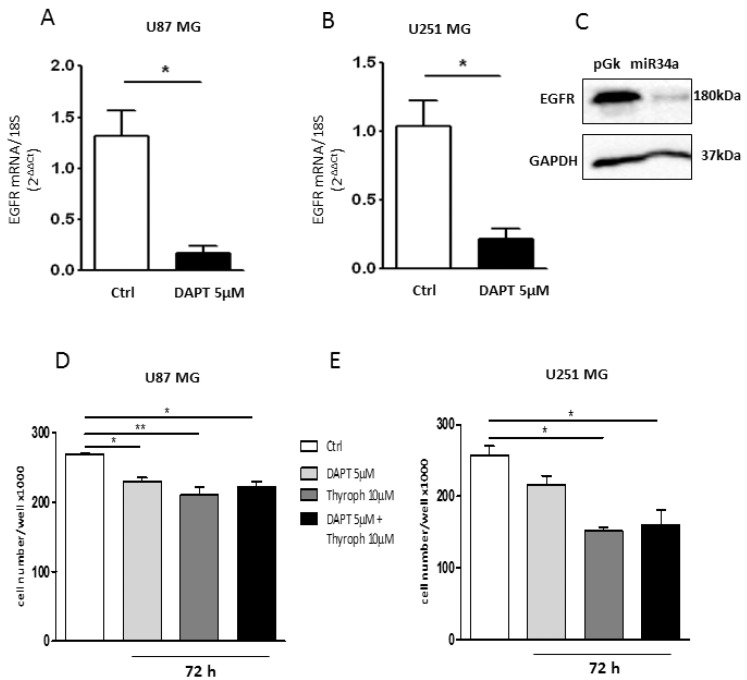
Effect of Notch/EGFR inhibition on GBM cell growth. Real time RT-PCR analysis of EGFR expression in U87MG (**A**) and U251MG (**B**) cells treated with 5 µM DAPT for 24 h; (**C**) Western blot analysis for EGFR in U87MG cells transfected with mir-34a-5p. GAPDH was used as the internal reference protein (* *p* < 0.05; *t*-test); (**D**,**E**). Analysis of the cell growth in U87MG and U251MG cells, respectively, treated with 5 µM DAPT, or 10 µM Tyrph or 5 µM DAPT plus 10 µM Tyrph (* *p* < 0.05; ** *p* < 0.01; *t*-test and one-way ANOVA test).

The Notch pathway is tightly coupled to EGFR signaling [10]. It has been demonstrated that Notch regulates EGFR expression in GBMs via p53 (both wild-type and mutated) [10]. To investigate whether in our cell models there was also a cross-interaction between Notch and EGFR, we treated both cell lines with 5 µM DAPT, a gamma-secretase inhibitor that prevents Notch cleavage and thus its activation [30]. Quantitative RT-PCR analysis demonstrated that DAPT downregulates the EGFR transcript in both U87MG (Figure 7A) and U251MG cells (Figure 7B) when compared to untreated cells. This result demonstrates that the inhibition of the Notch pathway also negatively modulates the EGFR expression in our cell models. In order to assess whether this effect was mediated by mir-34a-5p in U87MG cells, we measured the EGFR protein levels after ectopic expression of the miRNA. As shown in Figure 7C, a strong decrease of EGFR protein level was observed.

Finally, in order to correlate the downregulated expression of Notch/EGFR with cell proliferation, we evaluated cell growth in the presence of Notch (DAPT) and EGFR (Tyrphostin; Tyrph) inhibitors. As shown in Figure 7D,E, the inhibition of Notch activity by 5 µM DAPT significantly affected cell proliferation only in the U87MG cells. Conversely, the inhibition of EGFR activity by 10 µM Tyrph significantly reduced cell growth in both cell lines after 72 h of treatment. The combined treatment with both inhibitors did not produce a further decrease of cell growth, thus indicating that the EGFR pathway is more relevant for GBM cell proliferation.

## 3. Discussion

**Figure 8:**
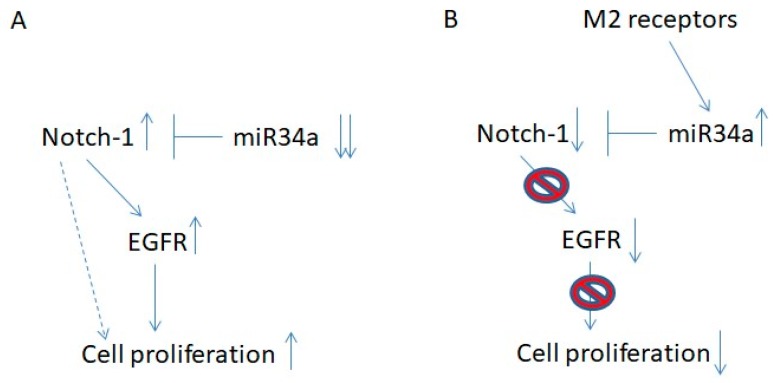
Schematic representation of the Notch-1/EGFR axis in U87 cells. (**A**) In U87 cells, the downregulated expression of mir-34a-5p caused the up-regulation of Notch-1 expression with consequent increased expression of EGFR. The up-regulation of these two receptors causes increased cell proliferation. (**B**) M2 muscarinic receptors up-regulate the expression of mir-34a-5p, which prevents the increased levels of Notch-1. The down-regulation of Notch-1 negatively affects cell proliferation by the down-regulated expression of EGFR.

Our previous data clearly demonstrated that M2 receptor activation, mediated by the agonist APE, induced a cytostatic effect on both GBM established U87MG and U251MG cell lines, impairing cell proliferation and inducing cell cycle arrest [17,18]. However, the molecular mechanisms underlying this cytostatic effect has not been explored. The present study revealed that it is achieved through different regulatory cascades, depending on the cellular context and mediated by several post-transcriptional regulators [31,32]. In particular, in the wild-type p53-expressing U87MG cell line, a relevant role is played by mir-34a-5p (Figure 8A). According to the tumor-suppressive role of this microRNA in different tumor histotypes [24,25,26,27], we found that its expression is strongly downregulated in GBM cell lines in comparison to the normal human brain (Figure 2). Interestingly the Notch expression appears directly correlated to mir-34a-5p expression; in fact the normal human brain, where the expression of mir-34a-5p is upregulated, the Notch-1 expression appears strongly reduced (Figure 2B). Instead in GBM cell lines, the down-regulated expression of mir-34-5p [21] correlates with Notch-1 increased levels.

In U87 cells, APE-treatment promotes p53 upregulation [18], which in turn causes the specific induction of mir-34a-5p and the following downregulation of Notch-1 and its downstream target, the transcription factor Hes-1. The downregulation of Notch-2, a direct target of mir-34a-5p in U87MG cells [28], was also observed, suggesting that the up-regulation of mir-34a-5p may impact on the expression of both Notch-1 and -2 receptor types. However, only in U87MG cells, which mimic wild type p53 GBMs, was the APE-treatment able to restore the tumor suppressor activity of mir-34a-5p (Figure 8B).

Differently, in the mutant p53-expressing U251MG cell line, the underlying molecular mechanism does not involve the regulatory activity of mir-34a-5p. Due to mutated p53, this miRNA is expressed to very low levels and is not induced in these cells after M2 agonist treatment (Figure 2A, Appendix A Figure A1). Notch-1 mRNA appeared increased by APE in U251MG cells, while protein levels were unaffected. However, this different expression may be dependent on post-transcriptional regulation where the mir-34a-5p appeared not to be involved. In fact, the significant decrease of Notch-2 mRNA and protein levels suggest that a transcriptional control or a post-transcriptional regulation mediated by other miRNAs may be involved.

Notch-1 and Notch-2 downregulation are however accompanied by a reduction of the downstream target gene Hes-1, confirming that the Notch pathway is affected both in U87MG and U251MG cells.

Notably, upon M2 receptor activation, a decrease of EGFR expression, which is upregulated in a large number of aggressive GBMs [9,33], has been observed in both cell lines. Notch-1, via p53, positively regulates EGFR transcription [10], therefore the downregulation of EGFR observed in U87MG cells could be explained through the miR34a-mediated Notch-1 downregulation (Figure 3D). Specific inhibition of Notch-1 activity, through either the gamma-secretase inhibitor DAPT or mir-34a-5p ectopic expression, produced the same effect on EGFR expression (Figure 7A,C). Different mechanisms may be invoked in the U251MG cell line, where mir-34a-5p is not induced (Appendix A Figure A1) and Notch-1 is not downregulated upon the M2 receptor activation (Figure 1D).

The analysis of cell growth in the presence of Notch and EGFR inhibitors highlighted that Notch inhibition affected cell growth particularly in U87MG cells (Figure 7D). The inhibition of EGFR activity significantly impaired cell growth in both cell lines. The co-treatment with both inhibitors did not show synergic effects, but the cell number was substantially unchanged with respect to the inhibition of EGFR activity (Tyrph) (Figure 7D,E).

Overall, these results suggest that EGFR is the main modulator of cell proliferation in GBM cells since its direct inhibition reduces cell growth in both the U87MG and U251MG cell lines while Notch may play a crucial role in the control of EGFR expression (Figure 8).

## 4. Materials and Methods

### 4.1. Cell Cultures

Human glioblastoma cell lines (U251MG and U87MG) (ATCC^®^ HTB-14™) were cultured in Dulbecco Modified Eagle Medium (DMEM, Sigma-Aldrich, St. Louis, MO, USA) supplemented with 10% fetal bovine serum (FBS) (Sigma-Aldrich, St. Louis, MO, USA), 50 µg/mL treptomycin, 50 IU/mL penicillin, 2 mM glutamine, 1% non-essential amino-acids (Sigma-Aldrich, St. Louis, MO, USA) and maintained at 37 °C, in an atmosphere of 90% air/10% CO_2_.

### 4.2. Pharmacological Treatments

At 24 h from seeding, cells were incubated in the presence of the M2 muscarinic receptor agonist arecaidine propargyl ester (APE) (100 µM) (Sigma-Aldrich, St. Louis, MO, USA) for different time points according to the experimental plan (24 h, 48 h). The selective binding of APE to M2 receptors has been previously demonstrated by pharmacological competition binding assay and M2 silencing experiments both in the GBM cell lines and in GB cancer stem cells [18,19,34].

GBM cells were also incubated for 24 h with 5 µM *N*-[*N*-(3,5-difluorophenacetyl)-l-alanyl]-(*S*)-phenylglycine *t*-butyl ester (DAPT) (Sigma-Aldrich, St. Louis, MO, USA), a gamma-secretase inhibitor or 10 µM *N*-(3-Chlorophenyl)-6,7-dimethoxy-4-quinazolinamine (Tyrphostin; Tyrph) (Sigma–Aldrich, St. Louis, MO, USA), as an inhibitor of EGFR activity.

### 4.3. Cell Viability

Cells were seeded onto a 24-well plate at a density of 1 × 10^4^ cells/well. After 24 h, cells were treated for 48 h with 5 µM DAPT, or 10 µM Tyrph. Cell growth was assessed by a colorimetric assay based on 3-(4,5-dymethylthiazol-2-y1)-2,5-diphenyltetrazolium bromide (MTT, Sigma-Aldrich, St. Louis, MO, USA) metabolization, according to Mosman [35]. For each well, the optical density (OD) at 570 nm was measured by a GloMax Multi Detection System (Promega, Madison, WI, USA).

### 4.4. Western Immunoblot

Cells were lysed in Laemmli Buffer (Biorad, Hercules, CA, USA) supplemented with 5% β-mercaptoethanol. Samples were heated for 5 min at 95 °C, loaded onto a 10% Tris-glycine polyacrylamide gel and run at 30 mA in a running buffer (25 mM Tris, 190 mM glycine, 0.08% (w/v) SDS). SDS-PAGE gels were transferred onto polyvinylidene fluoride (PVDF) membranes (Merck Millipore, Vimodrone, Italy) at 200 mA for 2 h in transfer buffer (20 mM Tris; 150 mM glycine, 10% (v/v) methanol). Membranes were blocked in 5% non-fat dry milk (MARVEL, Cambridge, UK) in 0.1% Tween-20 phosphate buffered saline (PBS) (Sigma-Aldrich, St. Louis, MO, USA) before incubation with the antibodies. The specific signal for each antibody was detected using an Enhanced Chemi Luminescence (ECL) kit (Immunological Sciences, Roma, Italy). The primary antibodies used were: (1) goat anti-Notch-1 (1:500) (Santa Cruz Biotechnologies, Santa Cruz, CA, USA), (2) rabbit anti-Notch-2 (1:200) (Santa Cruz Biotechnologies, Santa Cruz, CA, USA), and (3)mouse anti-EGFR (1:500) (Merck Millipore, Vimodrone, Italy). Rabbit anti-GAPDH (Abcam, Cambridge, UK) (1:2500) was used as the loading control. The HRP (horseradish peroxidase)–conjugated secondary antibodies (Sigma-Aldrich, Milan, Italy) were used at the 1:10,000 dilution in 5% (w/v) non-fat dried milk powder in T-TBS. The HRP signal was developed using the LiteAblot PLUS or TURBO extra sensitive chemiluminescent substrates (Euroclone, Milan, Italy).

### 4.5. RNA Extraction and Semi-Quantitative RT-PCR Analysis

Total RNA was extracted using the “Total RNA minikit” (GeneAid, New Taipei City, Taiwan) following the manufacturer’s instructions and digested with DNAse I (Ambion-Life Technologies Italia, Monza, Italy). Around 2 µg of RNA was reverse transcribed using random hexamers and M-MLV Reverse Transcriptase (Promega, Milano, Italy) and diluted to a final concentration of 20 ng/µL. The expression of Hes-1 transcript was evaluated by semi-quantitative RT-PCR analysis using the following primers: Hes-1, forward, 5′-ATGACAGTGAAGCACCTCCG-3′; reverse, 5′-AGGTCATGGCATTGATCTGG-3′. The 18S was used as housekeeping gene 18S, forward, 5′-CCAGTAAGTGCGGGTCATAAGC-3′; reverse, 5′-AACGATCCAATCGGTAGTAGCG-3′.

### 4.6. Real Time PCR Analysis

Around 100 ng of cDNA was used as the template for the real time RT-PCR reaction, using SyBRGreen Jump Start Taq Ready Mix (Resnova, Genzano di Roma, Roma, Italy) and the I Cycler IQTM Multicolor Real Time Detection System (Biorad, Hercules, CA, USA). Relative quantification was performed using the comparative ΔΔCT method [20,36].

The primers used were:

Notch-1: forward, 5′-AGGCATCATGCATGTCAAAC-3′;

reverse, 5′-TGTGTTGCTGGAGCATCTTC-3′

Notch-2: forward, 5′-TTGTGTGAACAATGGGCAGT-3′;

reverse, 5′TTCATAGCCATTCGGGTGAT-3′

Egfr: forward, 5′-AGCATGTCAAGATCACAGAT-3′;

reverse, 5′-TGGATCCAAAGGTCATCAA-3′;

18S: forward, 5′-CCAGTAAGTGCGGGTCATAAGC-3′;

reverse, 5′-AACGATCCAATCGGTAGTAGCG-3′

To analyze mir-34a-5p expression, cDNA was generated using the miScript II Reverse Transcription kit (Qiagen, Milan, Italy). qRT-PCR analyses were performed using the miScript-SYBR green PCR kit and specific DNA oligonucleotides by Qiagen on a 7500 Fast Real-Time PCR (Applied Biosystems Italia, Monza, Italy). Values obtained were normalized for snRNA U6.

### 4.7. Northern Blot Analysis

Specifically, 5 µg of total RNA from U87 cells untreated or treated with APE for 24 h was run on 10% polyacrylamide gel in 1× TBE, 7 M urea and transferred onto an Amersham Hybond-NX nylon membrane (GE Healthcare Italia, Milan, Italy). RNA cross-linking was performed in 0.16 M *N*-(3-Dimethylaminopropyl)-*N*′-ethylcarbodiimide hydrochloride and 0.13 M 1-methylimidazole (Sigma-Aldrich) at pH 8, for 2 h at 60 °C. DNA oligonucleotides complementary to the sequence of mature mir-34a-5p and 5S-rRNA (5′-AGACGAGATCGGGCGCGTTCA-3′) were ^32^P-labelled and used as probes. Densitometric analyses were performed using the Typhoon Imager and ImageQuant software (Molecular Dynamics, GE Healthcare, Little Chalfont, UK).

### 4.8. Expression Vectors and Transfections

Notch-1 3′UTR containing the miR-34a-5p putative binding site was amplified by PCR and cloned into Ψcheck2 plasmid (Promega Italia, Milan, Italy), downstream of the Renilla luciferase gene by using the following primers: forward, 5′-CCGCTCGAGCCGACCAGAGGAGCCTTTTTA-3′; reverse, 5′-TTTGCGGCCGCCTGTGTTGCTGGAGCATCTT-3′.

The same plasmid also contained the firefly luciferase gene to normalize for transfection efficiency. HEK293T cells were co-transfected with the control or miRNA over-expressing plasmids and wild-type Notch-1 3′UTR reporter plasmids. Cells were lysed after 48 h from transfection and luciferase activities were measured by using the Dual-Luciferase Assay Reporter System (Promega Italia, Milan, Italy) according to the manufacturer’s instructions.

### 4.9. Statistical Analysis

Data are representative of at least three independent experiments and are presented as mean ± SEM. Statistical analysis was performed by the Student’s t-test and one-way ANOVA followed by Tukey multiple comparison post-test. (* *p* < 0.05; ** *p* < 0.01; *** *p* < 0.001).

## 5. Conclusions

Our data showed for the first time, a cross-talk between the M2 muscarinic receptor and Notch and EGFR signaling pathways in GBM. This interplay relies on different regulatory cascades including miRNAs and depends on the cellular context and genetic background. In all cases, M2 receptors appeared to mediate onco-suppressor signals in GBM cells as already reported in other tumor types [37]. Moreover, the data emerging from our work also highlighted mir-34a-5p as a potential therapeutic tool for GBM cancer therapy considering its onco-suppressor function and reduced levels in the GBM cell line.
